# Modern Radiotherapy Technology: Obstacles and Opportunities to Access in Low- and Middle-Income Countries

**DOI:** 10.1200/GO.21.00376

**Published:** 2022-07-15

**Authors:** Priyamvada Maitre, Rahul Krishnatry, Supriya Chopra, Soehartati Gondhowiardjo, Beda Mnamala Likonda, Qazi Mushtaq Hussain, Eduardo H. Zubizarreta, Jai Prakash Agarwal

**Affiliations:** 1 Department of Radiation Oncology, Tata Memorial Centre, Mumbai, India; 2 Homi Bhabha National Institute, Mumbai, India; 3 Department of Radiation Oncology, Faculty of Medicine of Indonesia,Dr Cipto Mangunkusumo Hospital, Jakarta, Indonesia; 4 Bugando Medical Centre, Catholic University of Health Sciences, Nyamagana, Mwanza, Tanzania; 5 National Institute of Cancer Research and Hospital, Dhaka, Bangladesh; 6 Applied Radiation Biology and Radiotherapy Section, International Atomic Energy Agency, Vienna, Austria

## Abstract

Low- and middle-income countries (LMICs) have a large burden of cancer with differential population needs and outcomes compared to high-income countries. Access to radiotherapy, especially modern technology, is a major challenge. Modern radiotherapy has been demonstrated with better utility in overall cancer outcomes. We deliberate various challenges and opportunities unique to LMICs' set up for access to modern radiotherapy technology in the light of discussions and deliberations made during the recently concluded annual meeting of Tata Memorial Centre, India. We take examples available from various LMICs in this direction in our manuscript.

## INTRODUCTION

An estimated 19.3 million people worldwide were newly diagnosed with cancer in 2020, and 9.9 million died due to cancer.^1^ In the next 20 years, a steep rise of up to 30.2 million new cases and 16.3 million cancer deaths is estimated globally. However, increase in incidence and mortality is expected to be disproportionately higher in low- and middle-income countries (LMICs) as compared with high-income countries (HICs). In countries with high to very high Human Development Index, 43% increase in cancer incidence and 59% increase in cancer mortality are projected by year 2040.^2^ In contrast, incidence is estimated to be 72% higher and mortality 76% higher in countries with low to medium Human Development Index.^1^ Differential rate of population growth for LMICs and HICs partially accounts for higher cancer incidence and mortality in LMICs, as the average annual rate of population growth is estimated to be 0.9-2.0 in LMICs against 0.12 in HICs.^3^ This imbalance in the cancer burden is worsened by current disparities in resources to combat cancer, which need to be addressed on priority.

**CONTEXT**

**Key Objective**
Identifying obstacles and opportunities during implementation of modern radiotherapy technology in low- and-middle-income countries (LMICs).
**Knowledge Generated**
Summarized from global expert panel discussion in the annual meeting of Tata Memorial Centre (Evidence-Based Medicine 2021), this work is an overview of the unique challenges posed by evolving radiotherapy technology. We also examine the potential opportunities offered by technological advancements to improve access to quality radiotherapy in LMICs.
**Relevance**
LMICs must focus on sustainable investment strategies and locally adapted solutions to bridge the technological gap in radiotherapy. Resource allocation in national cancer care plans should harness the potential of technology to expand quantity and quality of radiotherapy services. Transition to modern technology at departmental, local, and national levels in LMICs should be facilitated by expert leadership and continued support for infrastructure and training.

Radiotherapy is a key resource against cancer with almost half of all patients with cancer requires radiotherapy as a part of their treatment. It is the mainstay of definitive treatment for advanced cancers of sites such as head/neck and cervix.^4,5^ These cancers are disproportionately higher in LMICs, in addition to being detected at more advanced, surgically inoperable stages. Hence, the requirement for radiotherapy in LMICs is even higher, for definitive as well as palliative therapy.^6^ Access to radiotherapy is therefore integral to cancer care planning in LMICs and needs to be addressed both in terms of quantity and quality. Rapid evolution in radiotherapy technology has impacted therapeutic management across cancer sites, but many barriers prevent the benefits of these advancements from reaching patients with cancer in LMICs.

The annual Evidence-Based Medicine meeting (EBM 2021) held at Tata Memorial Centre, Mumbai, examined the current challenges in accessing radiotherapy services in LMICs and discussed practical solutions implementable at various levels. In this article, we highlight the key obstacles and opportunities in implementing modern radiotherapy technology in LMICs, along with indigenous measures for using technology adapted to local needs.

## PRESENT REQUIREMENTS AND GAPS IN CAPACITY

Of the total 14,875 megavoltage radiation machines in operation globally, 9,365 (63%) are in HICs, 4,165 (28%) in upper middle-income countries, and only 1,342 (9%) in LMICs.^7^ This gap is more stark in terms of population served, as HICs account for < 20% of the global population. The International Atomic Energy Agency (IAEA) ideally recommends four radiotherapy units per million people, with a minimum of at least 1.5 units per million. For most LMICs, this figure is 0.01-1.0 per million at present, in contrast to 4-12 units per million in HICs.^7,8^ One radiotherapy unit is serving 0.12 million people in HICs, in contrast to 1 million in middle-income countries and five million in lower-income countries (LICs).^9^ However, rising cancer incidence has widened the gap between demand and supply of radiotherapy services in LICs in the past decade despite progress in radiotherapy utilization worldwide.^5^ For instance, 26 out of 54 African countries did not have any radiotherapy machine by 2020.^10^ Although radiotherapy capacity has increased in almost all African countries, relative coverage increased by only 2.7% from 2012 to 2020 because of higher increase in cancer burden (32%) relative to population growth (19%).^10^ Radiotherapy infrastructure in populous southern Asian LMICs is similarly limited. India with its current population of 1.36 billion has only 675 teletherapy machines (recommended 2040, shortfall 67%), 175 simulators (520, 66%), and 317 brachytherapy machines (650, 44%).^7,11^ Indonesia has only 82 teletherapy machines and 22 brachytherapy machines for 267 million population, with eight out of 34 provinces having no radiotherapy services and 10 provinces due to receive their first operational radiotherapy facility by 2023.^12^ The present rate of progress in capacity building, although encouraging, will clearly be inadequate to meet the estimated increase in radiotherapy demand. According to the Directory of Radiotherapy Centres, out of the 5,719 megavoltage units installed in the past decade, 8% were in LMICs versus 73% in HICs.^7^ This shortcoming needs to be addressed in a planned manner, and implementing cancer care plans at the national level has been associated with increase in the number of radiotherapy units acquired.^13^

With regards to the gap in technological quality of infrastructure, a significant proportion of the operational radiotherapy facilities have limited capacity to deliver conformal radiotherapy. Locally advanced cancers of head/neck, cervix, lungs, and breast constitute a large proportion of radiotherapy demand in LMICs. A comparison of mortality-to-incidence ratio for these cancer sites between HICs and LMICs clearly shows poor survival outcomes in majority of patients diagnosed in LMICs.^1^ Every year, 60,000 African women die out of the 80,000 diagnosed with cervix cancer, attributable to late diagnosis and lack of access to standard external beam radiotherapy (EBRT) and brachytherapy.^14^ Availability of basic standard radiotherapy facilities is a first step toward improving clinical outcomes for these patients. EBRT delivered by conventional techniques using telecobalt equipment still serve a sizable proportion of radiotherapy requirements in LMICs, up to 33% in LMICs like India in contrast to only 7% in HICs.^6,15^ Progress toward bridging the technological gap has been observed, with an IAEA update noting 78% increase in the number of linear accelerators across Africa.^10^ However, unsustainable procurement of advanced technology may potentially worsen its access where it is required the most. When the budgetary allocation toward radiotherapy is limited in most LMIC cancer care plans, the unmet radiotherapy may be better served by appropriate resource stratification. For instance, in regions with uncertain electricity supply and lack of trained personnel for quality control and maintenance, telecobalt equipment would be a better solution than high-end linear accelerators. Cost-effective modifications to existing equipment, such as incorporation of multileaf collimators to telecobalt machine for delivery of conformal radiotherapy, can be potential innovative solutions for optimizing available resources.

## TECHNOLOGICAL DISPARITIES, BARRIERS, AND MITIGATION STRATEGIES

Technological status of radiotherapy resources affects quality of clinical outcomes across cancer sites. Radiotherapy is indispensable for treatment of locally advanced cancers of breast, lungs, and head/neck region, which are projected to increase in LMICs by 70%-100%.^1^ Technological advancements in modern radiotherapy equipment have transformed the radiation delivery for these cancers over conventional telecobalt-based techniques.^16-21^ Major impact of modern technology has been toward reduction in treatment-related toxicity and improving treatment tolerance. In head/neck cancers, intensity-modulated radiotherapy (IMRT) reduces acute and late treatment-related morbidities including dysphagia, xerostomia, weight loss, trismus, fibrosis, and hearing loss.^18,19,22^ Studies in patients with head/neck cancer treated with curative-intent radiotherapy have also observed improved disease-free survival and overall survival with IMRT over cobalt-based conventional technique despite longer waiting time for IMRT.^23,24^ In gynecological cancers, the use of IMRT to deliver postsurgery radiotherapy reduces acute as well as late GI morbidity.^20,21^ For cervix cancer, one of the top three cancers in LMICs, three-dimensional (3D) volumetric brachytherapy, has shown improved local control and disease-free survival versus conventional two-dimensional (2D) point-based brachytherapy.^25^ Newer avenues such as ablation of oligometastatic lesions in brain, lungs, liver, and bones have been opened by technological advancements in targeted radiotherapy, which may be more cost effective than chemo/systemic therapy.^26,27^ Highly focused proton beams are improving upon morbidity of pediatric radiotherapy.^28^ Quality of survivorship has certainly improved with advanced radiotherapy, although this effect has been largely limited to HICs. For LMICs to allocate their limited resources toward expensive technology, its benefits must extend beyond lowering patient-level morbidity.

Toxicity-sparing potential of advanced conformal radiotherapy techniques should be harnessed for shorter schedules of hypofractionated radiotherapy in various tumor sites, to widen the reach of radiotherapy within LMICs. Shifting from conventionally fractionated standard schedules of radiotherapy toward hypofractionated schedules would lead to considerable savings on patients' indirect expenses of travel and lodging. At the population level, it helps expand access to radiotherapy by allowing more patients to be treated within the same period. A simple calculation for a linear accelerator operating 12 hours a day for 5 days a week (total 60 hours a week) and treating four patients per hour (48 patients per day or 240 fractions per week) shows that per week, 48 patients can be treated with five-fraction schedule or 16 patients with 15-fraction schedule, against only nine patients with 25-fraction schedule. A collaborative cost analysis study used the IAEA Radiotherapy Cost Estimator tool to calculate the potential cost savings if hypofractionated schedules were used for breast and prostate cancer in African countries. It estimated potential maximum savings of up to $1.1 billion US dollars (USD) and $606 million USD for breast and prostate radiotherapy, respectively, and radiotherapy access was projected to increase by 0.3%-36% by full implementation of hypofractionation.^29^ Economic sustainability of hypofractionated schedules have been demonstrated in several cost-benefit analyses^27,30,31^ and also being explored in randomized trials (ClinicalTrials.gov identifier: NCT03561961).^32^ The costs of technology-intensive radiotherapy can be prohibitive at the individual and public level in LMICs, but growing experience with hypofractionation has gradually made it technologically less demanding. Many of the shorter schedules are deliverable now with midrange linear accelerators, representing an opportunity to deliver optimal radiotherapy at lower costs and bridge the growing radiotherapy requirements in LMICs.

Multiple barriers restricting access to high-quality radiotherapy technology have been identified across HICs and LMICs. The pan–European Health Economics in Radiation Oncology project identified various patient factors (age, comorbidities, lower awareness), physician factors (referral bias, physician preferences), and geographical factors (distance of facility from residence, imbalanced geographical distribution of treatment centers) as significant factors contributing to lower utilization rates of IMRT/image guided radiotherapy in the European nations.^33^ Additional limitations in infrastructure, especially in relation to Eastern versus Western European nations, were also observed. For instance, about 40% of the megavoltage radiotherapy machines in Eastern Europe are telecobalt units, not capable of delivering modern conformal radiotherapy.^34^ In LMICs, the most significant barrier to access is the imbalanced distribution of the limited resources. Most LMICs including India have low allocation of gross domestic product (GDP) spending toward health care, despite the enormous economic losses of cancer mortality/morbidity. Cancer burden accounted for as high as high as 0.49% of GDP lost for South Africa and 0.36% of GDP lost for India.^35^ In contrast, the total health care spend was only 1.29% of GDP in India in 2019-2020, inadequate to achieve the goal of equitable access to modern cancer care. A costing study for head and neck cancers from a tertiary cancer center in India estimated that the health care cost of treating a patient with conformal radiotherapy was 3-9 times higher than for the conventional telecobalt machine, and the radiotherapy planning cost was 4.3-5.5 times higher for 3D versus 2D treatment.^36^ Given the unbalanced investment in private and public (governmental) sector, most of the advanced radiotherapy facilities in LMICs are privately controlled. This creates a monetary barrier to access of modern radiotherapy technology. Another barrier is geographical due to urban concentration of cancer treatment centers. For instance, 40% of top cancer centers in India are located in major metropolitan cities, and 85% are under non–governmental ownership.^37^ In countries with large area such as India, considerable state-level disparities in availability of EBRT and brachytherapy infrastructure directly impact patients' access to standard of care radiotherapy.^11^

Awareness of barriers to technological access at all the levels of policy- and decision-making is required, along with clarity on understanding the value of high-quality radiotherapy. Active participation of clinical experts in policy-defining bodies and ground-level estimation of the present and future technological needs is essential for achieving the desired access to quality radiotherapy. Furthermore, investment policies must account for upgradation of radiotherapy technology includes the cost of procurement of new equipment in addition to expenses of infrastructure building, salaries of trained personnel, and more person-hours for operation. Maintaining a quality brachytherapy service has to factor in the expense of timely renewal of brachytherapy source and maintaining inventory of advanced applicators. Quality assurance of radiotherapy equipment and processes needs to be more rigorous with advancement in technology. These technical aspects require constant engagement with experts and professional bodies, which is often deficient in LMICs. Besides, lack of awareness among health care providers is another major obstacle for patients in accessing radiotherapy. The national guidelines have been formulated for major cancers in many LMICs such as India and Indonesia, but referral of patients requiring radiotherapy to appropriate facilities is inadequate.^12,38^ This reduces the clinical effect of cancer treatment and is consequently associated with additional expenses of further salvage therapies.

## VALUE OF INVESTMENT IN MODERN RADIOTHERAPY TECHNOLOGY

The total number of patients with cancer with indications for radiotherapy (defined as clinical situations where radiotherapy would improve survival) is estimated to rise from 7 million to 12 million by 2035. Optimal radiotherapy access and utilization can provide 2.5 million local controls and save 1 million lives annually by 2035.^2^ Yet, the value of investment in radiotherapy is seldom realized at the policy level in terms of economic costs. The Lancet commission on radiotherapy access estimated the cost of radiotherapy scale-up across all LMICs to be $96.8 billion USD using the efficiency model. By 2035, this would save an astounding 26.9 million life-years over the lifetime of patients treated in LMICs, producing an economic benefit of $365.4 billion USD and returns of $104.2 billion USD by the human capital approach.^39^

Modern technology of intensity modulation, image guidance, stereotactic ablation, and integration of radiological and functional imaging in radiotherapy planning has changed the face of radiation oncology as an integral pillar of cancer treatment. Investment in radiation technology will have to account not only for setting up new modern radiotherapy facilities but also for the cost of upgradation of existing units to meet the current technological standards. Policymakers in LMICs must be made aware of the huge potential return on investment in upgraded radiotherapy technology, with regards to the long-term economic gains as well as quality of life. To realize this potential, a shift is needed in the perspective away from short-term gains on investment toward the eventual goal of improving quality of cancer care. To illustrate, an Indian study in patients with cervical cancer compared cumulative income over 5 years for women treated with magnetic resonance imaging–based image-guided brachytherapy (MR-IGBT) versus conventional radiograph–based brachytherapy. It estimated that treating patients with MR-IGBT instead of conventional radiograph–based brachytherapy would generate a simulated excess income of Indian Rupee (INR) 4-45 million over 5 years, which could be up to 66% of the expenses required to practice exclusive MR-IGBT.^40^ Another study estimated that the cost of managing late radiation toxicity in patients with cervical cancer could be almost equivalent to the cost of primary treatment itself, highlighting the importance of using advanced technology to minimize treatment morbidity.^41^

Multiple cost-effectiveness analyses have explored the incremental cost effectiveness ratio of the cost of gain in per quality-adjusted life-year (QALY) by implementation of IMRT and stereotactic body radiotherapy across cancer sites. IMRT in postoperative gynecological radiotherapy has shown significant reduction in late toxicity as compared with three-dimensional radiotherapy while maintaining clinical outcomes.^21^ Although IMRT was not cost-effective during early toxicity phase, over next few years of follow-up, it became cost-effective per QALY gained because of lower toxicity costs.^42^ Similarly, studies for prostate cancer and oropharyngeal cancers showed that despite the higher upfront treatment costs with IMRT, reduction in late toxicity made IMRT more cost-effective.^43,44^ The incremental cost-effectiveness ratio with technologically advanced techniques of IMRT and IGBT has been observed to be about $5,000-30,000 USD per QALY gained, much below the generally accepted threshold of $50,000 USD by the insurance payers in HICs.^43-45^ A cost-effectiveness study for patients with head and neck cancer in India estimated an incremental cost of $7,072 USD and $5,164 USD per QALY gained compared with 2D and 3D conformal radiotherapy, respectively.^46^ Evidently, investment in radiotherapy technology yields much higher gain in QALY for patients as compared with many other treatment modalities. Given the proof of cost-effectiveness of advanced EBRT and brachytherapy techniques, widening of patients' access to these facilities needs to be prioritized.

It must be emphasized that out of all the modalities of cancer care, radiation is the least site-specific. Investment in upgradation of radiation delivery techniques is likely to have the broadest impact across treatment of major cancers. With improving income levels across LMICs, the epidemiological shift in cancer sites would bring increased burden of cancers of breast, lung, prostate, and digestive tract. Radiotherapy across these sites has already transitioned toward technology-intensive ablative schedules, covering even the oligometastatic spectrum to enhance quantity and quality of survival. This would mean increased proportion of cancer survivors, with burden of late toxicity and risks of secondary cancers. Not to forget, the absolute numbers of patients with cancer will remain high even for cervix and head/neck cancers because of rising cancer incidence in population. Investment in radiotherapy technology in LMICs will need to account for both these factors to bear the burden of cancer care for next few decades. The Global Task Force on Radiotherapy for Cancer Control model estimated that the investments in developing radiotherapy capacity could be expected to recoup within 10-15 years.^47^ Therefore, investment in modern radiotherapy technology is a value proposition for reducing the burden of cancer morbidity and translating it into economic benefits.

## OPPORTUNITIES AND SOLUTIONS

The value of radiotherapy as an effective, life-saving treatment for cancer has been demonstrated conclusively. To enable access to its full benefit for the patients with cancer, first step is to generate reliable estimates of requirement, both present and future, at a national level to establish the unmet need. Existing lacunae and desired goals must be identified and defined clearly in objective terms (Table 1). As a first step, cancer should be delinked from other noncommunicable diseases in the LMIC health care programs and covered under mandatorily notifiable diseases. It would generate more reliable estimate of cancer burden and help in prioritizing common cancer sites for action. Population-based cancer registry data are deficient and skewed toward urban population in LMICs like India, where only 2 out of 28 population-based cancer registries are dedicated to rural population.^48^ Cancer mortality in LMICs is also underestimated because of deficient identification of cancer as a cause of death, for which verbal autopsy-based projection estimates have highlighted preventable causes of cancer.^49,50^ More accurate estimation of cancer burden would be essential to generate ground-level evidence of infrastructure requirements.

**TABLE 1:**
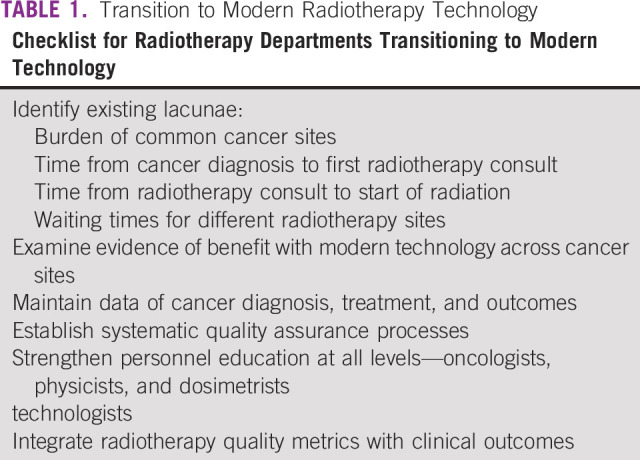
Transition to Modern Radiotherapy Technology

The challenge of deficient infrastructure is a potential opportunity for radiotherapy vendor companies to expand into hitherto untapped markets. The total number of megavoltage radiotherapy units by year 2035 needs to be nearly quadrupled from 1,303 to 5,200 in LMICs.^7,39^ Governments in LMICs may leverage economies of scale to their advantage to negotiate buying and maintenance of new equipment. In addition, local solutions for manufacturing radiotherapy accessories such as immobilization devices can add to cost savings. For instance, Department of Atomic Energy in India has supported the local production of radiotherapy equipment and devices at a fraction of global costs. Collaborations between global and indigenous entities for manufacture, installation, operation, and maintenance of radiotherapy infrastructure should be incentivized to establish models of sustainable investment. Innovations in technology focusing on improved efficiency and adaptability should be aimed at, such as less complex single-energy linear accelerators optimized for longer hours of operation and resilient in unreliable power supply conditions.

In LMICs, investment is majorly required for establishment of new radiotherapy facilities as well as upgradation of existing facilities. After the primary infrastructure is set up, the recurring costs of machine maintenance have to be accounted for as well. Rather than publicly funded and private centers competing against each other for investment, a model of public-private partnership may be more sustainable, with government assuming a largely regulatory role. Build-operate-transfer scheme of public-private partnership is a feasible option for investment in modern technology. This scheme of allowing private sector to install brachytherapy and tomotherapy in Indonesia has been successfully implemented in a government hospital in Indonesia.^12^ Encouragingly, cancer care is increasingly being prioritized for resource allocation within the health care plans in many LMICs. In India, the Tertiary Cancer Care Centers (TCCCs) scheme under the National Program for Prevention and Control of Cancer, Diabetes, Cardiovascular Diseases, and Stroke aims at setting up and strengthening of 20 State Cancer Institutes and 50 TCCCs for providing comprehensive cancer care in the country.^51^ The scheme provides for a one-time grant of INR 1.2 billion per state cancer institute and INR 0.45 billion per TCCC for building construction and procurement of equipment. Yet, a report by the Federation of Indian Chambers of Commerce & Industry estimates investment requirement of about INR 250 billion to meet the radiotherapy needs of new patients with cancer over next 5 years.^52^

State-sponsored health insurance schemes for patients with cancer would also be critical for negotiating the existing financial barriers between public and privately owned facilities. As an example, Aayushman Bharat and Pradhan Mantri Jan Arogya Yojana schemes by the Government of India reimburse the complete cost of advanced radiotherapy services for insurance holders to get treated at private hospitals, subject to a capped limit. This solution improves access to quality cancer care for economically disadvantaged sections. Its eventual success would depend on creating a large enough base of beneficiaries, to make it economically attractive for private centers to participate in these schemes.

Traditional health care systems in most LMICs have to be adapted or shifted toward a more standardized and quality-metric–based approach of modern cancer care. The hub-and-spoke health care model is highlighted as an alternative for more efficient utilization of resources. This approach is being favored by the National Cancer Grid initiative of Government of India, with establishment of 50 TCCC supplemented by strong regional cancer centers at state levels.^53^ It envisages a system of providing continued support and guidance to smaller centers from national and global experts. It also provides for establishment of telecommunication networks for standardizing cancer management, launching education initiatives, creating a trained radiotherapy workforce, and collaborating for national-level cancer research. The long-term goal was to strengthen the preventive and screening measures to reduce the burden of advanced cancer while simultaneously scaling up the therapeutic facilities to improve delivery of quality cancer care. Similarly, the African Radiation Oncology Network established by the IAEA has connected radiation oncology peers throughout Africa for telemedicine-based case discussions and educational activities, strengthening clinical decision-making and resident education.^54^

**FIG 1:**
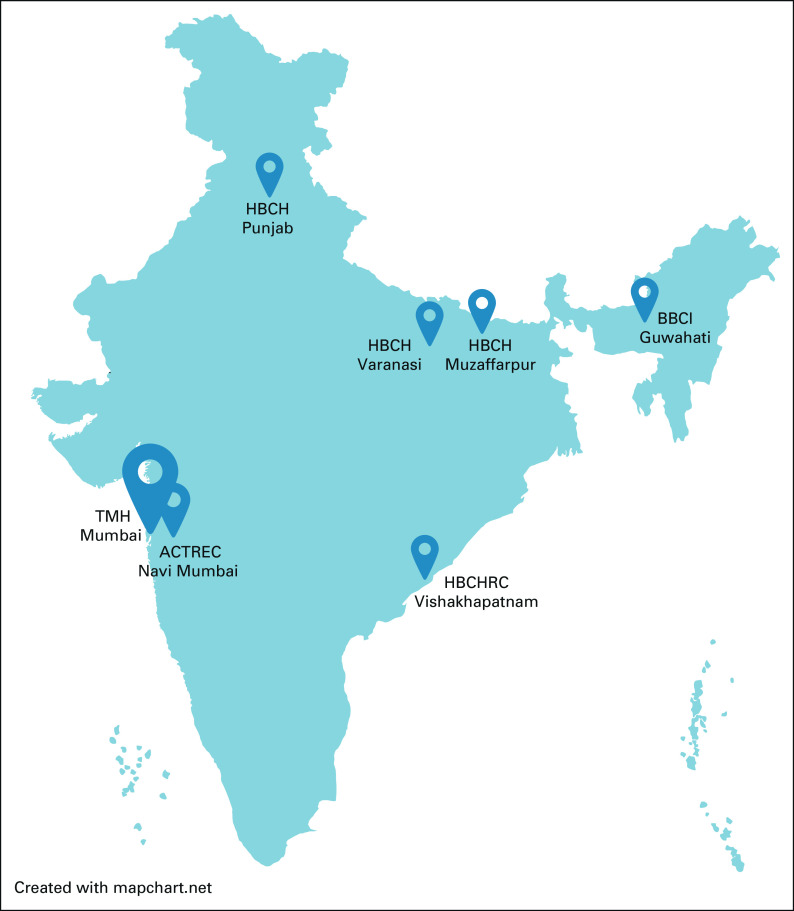
Hub-and-spoke model of cancer care at Tata Memorial Centre, India. ACTREC, Advanced Centre for Treatment, Research and Education in Cancer; BBCI, Bhubaneswar Borooah Cancer Institute; HBCH, Homi Bhabha Cancer Hospital; HBCHRC, Homi Bhabha Cancer Hospital & Research Centre; TMH, Tata Memorial Hospital.

For improved understanding of relevant barriers and potential solutions, participation of local-level researchers is essential. Research into the challenges to radiotherapy access in LMICs would flourish only with meaningful on-ground collaboration of native clinicians and experienced global public health leaders. Developed nations and global cancer care organizations can contribute by highlighting the local voices in academic collaborations and publications. In addition, education exchange programs between mentors in leading cancer institutes and mentees in LMICs are valuable in building up a trained workforce, who can then disseminate these skills to lower levels. Rather than the top-down approach from HICs to LMICs, the impetus toward upgradation of radiotherapy technology must come from national institutions who are better placed to implement proposed solutions. To illustrate as an example, Tata Memorial Centre in India is leading the mission of accessible cancer care in various parts of India under the aegis of National Cancer Grid initiative. The main center in Mumbai is providing administrative, financial, infrastructural, and educational support to smaller branch centers in underserved regions of India (Fig 1). These centers are technologically linked to the main institution with common interfaces for electronic medical records, medical imaging archives, and radiotherapy information portals for integration and standardization of treatment practices. With sustained support from the main hub to overcome operational hurdles, these spokes are envisaged to become regional hubs of quality cancer care over time.

To summarize, implementation of modern radiotherapy technology in LMICs not only poses unique challenges but also provides novel opportunities for improving access to cancer care by bridging geographical barriers. Caution is suggested against a few potential pitfalls while addressing the gaps in radiotherapy access (Table 2). Consolidated action toward prioritizing access to quality radiotherapy services is imperative at all levels of cancer care strategy for improving the quantity as well as quality of survival for patients with cancer in LMICs.

**TABLE 2:**
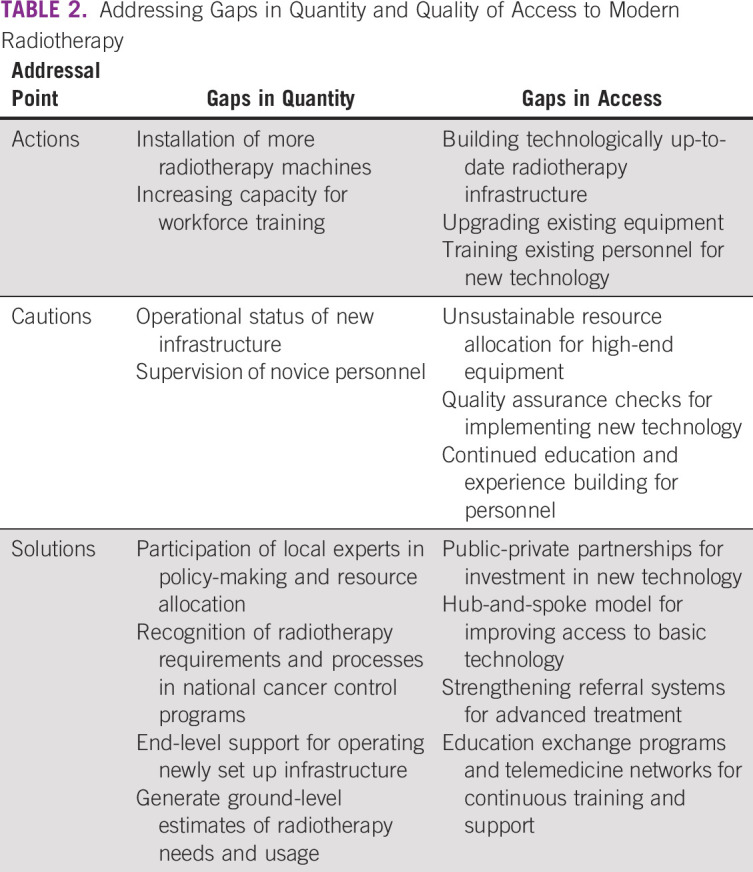
Addressing Gaps in Quantity and Quality of Access to Modern Radiotherapy
